# Short-Term Outcomes of Hip Fractures in Patients Aged 90 Years Old and Over Receiving Surgical Intervention

**DOI:** 10.1371/journal.pone.0125496

**Published:** 2015-05-15

**Authors:** Wei-Ting Lin, Chien-Ming Chao, Hsuan-Chih Liu, Yi-Ju Li, Wei-Jing Lee, Chih-Cheng Lai

**Affiliations:** 1 Department of Orthopedics, Chi Mei Medical Center, Tainan, Taiwan; 2 Department of Physical Therapy, Shu Zen Junior College of Medicine and Management, Kaohsiung, Taiwan; 3 Departments of Intensive Care Medicine, Chi Mei Medical Center, Liouying, Tainan, Taiwan; 4 Department of Nursing, Min-Hwei College of Health Care Management, Tainan, Taiwan; 5 Department of Emergency medicine, Chi Mei Medical Center, Tainan, Taiwan; Uppsala University, SWEDEN

## Abstract

**Background:**

The knowledge about short-term outcomes of nonagenarians undergoing surgery for hip fracture in Asian is limited.

**Methods:**

The patients with hip fractures who underwent hip hemiarthroplasty and open reduction with internal fixation (ORIF) for management during the period from 2008 to 2012 were identified and their medical record was retrospectively reviewed.

**Results:**

During the study period, a total of 101 patients underwent surgery for management of hip fractures, and the age of patients ranged from 90 to 96 years. The sites of hip fracture were intertrochanteric (n = 57, 56.4%) and the neck of the femur (n = 44, 43.6%). Most of the patients had American Society of Anesthesiologists scores of 3(n = 55) or 4 (in 44 patients). 80.2% (n = 81) underwent the operation within one day after admission; however, there were 13 patients (12.9%) that underwent surgery 48 or more hours later. ORIF and hemiarthroplasty were performed for 63 (62.4%) and 38 (37.6%) patients, respectively. Overall, the 30-day and 1-year mortality rates were 9.9% (10/101) and 17.3% (13/75), respectively. Multivariate analysis showed that the 30-day mortality was significantly associated only with end-stage renal disease (ESRD) (Odds ratio, 11.13, 95% confidence interval, 1.275–97.881, P = .029).

**Conclusions:**

The short-term outcome of surgical management for Asian nonagenarians with hip fractures is favorable in selected patients.

## Introduction

The aging population is rapidly increasing. By 2050, the population who are 65 or more years of age is estimated to be about 1.5 billion, representing 16% of the global population by WHO estimation. Therefore, modern medicine will need to care for an increasingly ageing population in this era of aging. Hip fracture is one of the most common clinical diseases among elderly patients because they are more often osteoporotic and more likely to fall than younger people [1, 2]. In Taiwan, a nationwide population-based study showed that the annual incidence of hip fracture in the elderly population increased significantly from 1996 to 2002, and the average annual incidence of patients older than 85 years was 9.9 times higher than that of women 65 to 69 years of age and 7.9 times higher than that of men the same age [3]. At present, hip fracture is managed by open reduction with internal fixation (ORIF), hemiarthroplasty, or total hip replacement (THR), according to the degree of fracture displacement, functional requirements, patient age, comorbidity, and risk factors for surgery and anesthesia. Although THR provides better functional recovery, lower reoperation rates, and greater cost-effectiveness than ORIF and hemiarthroplasty [4–6], it is a complicated issue in extremely elderly patients. In Taiwan, THR is not routinely performed for elderly patients with hip fracture because of concerns of non-payment by National Health Insurance Administration. Therefore, physicians routinely perform non-THR surgical intervention for extremely elderly patients with hip fractures. Among patients over the age of 90 years, surgical intervention for hip fracture should be carefully considered due to concern regarding old age and multiple comorbidities. However, studies of surgical intervention for hip fractures among nonagenarians are limited, and most of them were performed in western countries [7–12]. Therefore, the object of this study is to investigate the short-term outcomes of nonagenarians undergoing surgery for hip fracture in Taiwan.

## Methods

### Hospital setting and patient selection

This study was conducted at the Chi Mei Medical Center, a 1300-bed hospital located in southern Taiwan. Patients who were older than 90 years and who had undergone surgery for hip fracture during the period from 2008 to 2012 were identified from the hospital’s computerized database. The medical records of all of the identified patients were retrospectively reviewed. Information regarding age, gender, type of fracture, underlying diseases, and the timing and types of surgery were recorded. The data was collected on a routine basis and the analysis was carried out retrospectively. Therefore no informed consent was required and it was specifically waived by Institution Review Board of Chi Mei Medical Center. Ethics approval was obtained from Institution Review Board of Chi Mei Medical Center.

### Definitions

The American Society of Anesthesiologists (ASA) physical status classification was used to evaluate the suitability of patients before surgery and includes six categories: 1) healthy, 2) mild systemic disease, 3) severe systemic disease, 4) severe systemic disease that is a constant threat to life, 5) a moribund person who is not to expected to survive without the operation, and 6) a brain-dead person whose organs are being removed for donor purposes. To investigate the clinical impact of different surgeries, we divided the surgeries into two primary types: 1) hemiarthroplasty (Austin Moore cemented hemiarthroplasty) and 2) ORIF (dynamic hip screw, DHS), reconstruction nail, cannulated screws). To evaluate the effectiveness of the timing of surgery, we classified the patients into two subgroups: 1) patients who underwent surgery within 24 hours of admission, and 2) patients who underwent surgery 24 hours or more after admission.

### Outcomes

To evaluate the clinical outcomes of patients, the length of hospital stay, 30-day mortality rate, and readmission rate were evaluated. In addition, postoperative complications, including any medical or surgical adverse events occurring following surgery, were retrospectively identified. All of the complications and the mortalities were identified according to the medical record during the follow-up in our hospital.

### Statistical analysis

Continuous variables are expressed as means ± standard deviations. These variables were compared using the Wilcoxon rank sum test or Student’s independent *t* test, as appropriate. Categorical variables were compared using the chi-square test or Fisher’s exact test. A *P* value < .05 was considered to show statistical significance. A multivariate forward logistic regression model was used to identify risk factors for mortality. All statistical analyses were conducted using the statistical package SPSS for Windows (Version 19.0, SPSS, Chicago, Il, USA).

## Results

### Clinical characteristics

During the study period, a total of 101 patients underwent surgery for management of hip fractures and their clinical features were summarized in Table 1. In addition, there are a total of 113 nonagenarians with hip fracture who were not treated surgically due to varied reasons during the study period. The age of patients ranged from 90 to 96 years (mean, 91.9 years) and women compromised most of the patients. The sites of hip fractures were intertrochanteric (n = 57, 56.4%) and the neck of the femur (n = 44, 43.6%). Hypertension was the most common underlying disease, followed by diabetes mellitus. Most of the patients had ASA scores of 3(n = 55) or 4 (n = 44); two patients had ASA scores of 2. Of the 101 patients undergoing surgery, 80.2% (n = 81) of them underwent surgery within 24 hours of admission; however, 13 patients (12.9%) underwent surgery 48 or more hours after admission. During surgery, about 75% of patients received a blood transfusion. DHS was the most common type of device (n = 53, 52.5%) followed by Austin Moore cemented hemiarthroplasty (n = 38, 37.6%), cannulated screws (n = 5, 5.0%), and reconstruction nails (n = 5, 5.0%) (Table 2). General anesthesia was the most common type of anesthesia in 87 patients, followed by spinal anesthesia.

**Table 1:** Demography of patients.

Variable	No. (%) of patients (n = 101)
Age, yr (mean ± SD)	91.9 ± 1.9
Female ratio	76 (75.2)
Site of hip fracture	
Intertrochanter	57 (56.4)
Femoral neck	44 (43.6)
Hemoglobin, g/dl (mean ± SD)	11.3 ± 1.7
Underlying diseases	
Hypertension	50 (49.5)
Diabetes mellitus	18 (17.8)
Coronary artery disease	14 (13.9)
Congestive heart failure	14 (13.9)
Chronic obstructive pulmonary disease	13 (12.9)
Cancer	12 (11.9)
Stroke	9 (8.9)
End-stage renal disease	4 (4.0)
ASA scores (mean ± SD)	3.4 ± 0.5
Time to operation after admission	
< 24 hours	81 (80.2)
24–48 hours	7 (6.9)
> 48 hours	13 (12.9)
Transfusion during operation	76 (75.2)
Outcomes	
Major complication	7 (6.9)
Length of hospital stay, days (mean ± SD)	12.4 ± 17.0
30 days readmission rate	16 (15.8)
30-day mortality rate	10 (9.9)
One-year mortality (n = 75)*	13 (17.3)

*Only 75 patients followed up one year after surgery

**Table 2:** The fixation method and device method based on the fracture type.

Fixation methods	Number (%) of each types of hip fracture		
	Intertrochanteric fracture (n = 57)	Femoral neck fracture (n = 44)	Total (n = 101)
Hemiarthroplasty			
Austin Moore cemented hemiarthroplasty	1	37	38 (37.6)
Open reduction and internal fixation			
Dynamic hip screw	51	2	53 (52.5)
Cannulated screws	1	4	5 (5.0)
Reconstruction nail	4	1	5 (5.0)

### Outcome analysis

The average of length of hospital stay was 12.4 days. Seven patients developed complications after surgery, and pneumonia was the most common type of complication. In addition, 16 patients had readmissions within 30 days of discharge. Among the 96 patients who had available records of activities of daily living (ADL) after surgery, 39 patients remained bedridden, 39 patients had wheelchair ambulation activities, 16 patients had walker ambulation activity and two patients had cane ambulation activity (Table 3). Overall, the 30-day and 1-year mortality rates were 9.9% (10/101) and 17.3% (13/75), respectively. The causes of death within 30 days were pneumonia (n = 6) and acute myocardial infarction (n = 4). The mean survival duration between surgery and mortality among these ten patients was 14.9 days.

**Table 3:** Functional status before and after-operation of patients who had available records of activities of daily living.

Functional status	Number (%) of patients	
	Before operation (n = 85)*	After operation (n = 96)**
Ambulation smoothly	51 (60.0)	0 (0.0)
Cane ambulation activity	11 (12.9)	2 (2.1)
Walker ambulation activity	7 (8.2)	16 (16.7)
Wheelchair ambulation activities	14 (16.5)	39 (40.6)
Bedridden	2 (2.4)	39 (40.6)

*Only 85 patients who had available records of activities of daily living before operation period

**Only 96 patients who had available records of activities of daily living after rehabilitation during post-operation period

Table 4 shows the comparisons of clinical manifestations between patients who survived and those who died within 30 days. Multivariate analysis showed that the 30-day mortality was significantly associated only with underlying end stage renal diseases (ESRD) (Odds ratio, 11.13, 95% confidence interval, 1.275–97.881, *P* = .029). In addition, we found that risk of 30-day mortality was not associated with age, gender, underlying illness or disease, site of fracture, or type and timing of surgery.

**Table 4:** Comparison between patients who survived and patients who died within 30 days after surgery.

Variable	No. (%) of death (n = 10)	No. (%)of survivor (n = 91)	P-value
Age, yr (mean ± SD)	91.9 ± 2.3	91.9 ± 1.9	0.944
Female ratio	8 (80.0)	68 (74.7)	0.715
Site of hip fracture			0.090
Femoral neck	7 (70.0)	37 (40.7)	
Intertrochanter	3 (30.0)	54 (59.3)	
Hemoglobin, g/dl (mean ± SD)	10.8 ± 1.3	11.4 ± 1.7	0.318
Comorbidities			
Hypertension	5 (50.0)	45 (49.5)	0.974
Congestive heart failure	3 (30.0)	11 (12.1)	0.135
Cancer	3 (30.0)	9 (9.9)	0.079
End stage renal disease	2 (20.0)	2 (2.2)	**0.024**
Stroke	2 (20.0)	7 (7.7)	0.213
Coronary artery disease	2 (20.0)	12 (13.2)	0.557
Diabetes mellitus	1 (10.0)	17 (18.7)	0.504
Chronic obstructive pulmonary disease	1 (10.0)	12 (13.2)	0.811
ASA scores (mean ± SD)	3.4 ± 0.5	3.4 ± 0.5	0.922
Time to operation			0.410
≤ 24 hours	7 (70.0)	74 (81.3)	
> 24 hours	3 (30.0)	17 (18.7)	
Transfusion during operation	9 (90.0)	67 (73.6)	0.279
Management			0.136
Hemiarthroplasty	6 (60.0)	32 (35.2)	
Open reduction and internal fixation	4 (40.0)	59 (64.8)	

## Discussion

We investigated the short-term surgical outcomes of nonagenarians with hip fractures in Taiwan due to the lack of published information on this age group. In Taiwan, most medical payments are regulated by the National Health Insurance program, and THR is not recommended for nonagenarians with hip fractures, based on NHI regulations. Therefore, non-THR surgical intervention is the most common option for treatment of elderly patients with hip fractures. The present work should provide a unique picture about the clinical outcomes of Taiwanese nonagenarians with hip fractures who undergo non-THR surgery. First, the 30-day mortality rate was only about 10% among the nonagenarians in our study. In the UK, a prospective observational study of 146 patients over the age of 90 years who were suitable for surgical fixation of their hip fractures showed that the overall mortality rate within 30 days was 14.4% (n = 21) [10]. Another study of 58 patients over 90 years of age in the UK showed that there was no postoperative death within 30 days after surgery [12]. Although our study may be different from other studies in design, population, and the type of management, the low mortality rate in the present work is similar as previous studies in western countries [10, 12]. Overall, it suggests that surgery may be a safe procedure for nonagenarians with hip fractures.

The causes of the 10 deaths in this study included infection and a cardiovascular event. This finding is consistent with previous studies of etiologies of postoperative mortality among elderly patients, which included pneumonia, myocardial infarction, and cerebral vascular accident [10, 12]. This should remind us that particular attention should be taken to prevent infections and cardiovascular events during routine postoperative care to avoid such fatalities. The strategies, such as elevation of head of the bed at least to 30 degree at day time, chest care, sitting training, and early rehabilitation after surgery, may be helpful for reducing these complications.

By multivariate regression analysis, we identified that ESRD was the only independent risk factor associated with postoperative mortality. It is plausible that ESRD itself could increase the risk of infection and cardiovascular complications [13–18], which were the two main causes of postoperative mortalities in this study. In one study of 13 ESRD patients whose age ranged from 54 to 77 years and who underwent surgical treatment for hip fractures [19], the overall mortality at 1 and 2 years was 23% and 30%, respectively. Another study of 18 hemodialysis and renal transplant patients who underwent total hip arthroplasty [20] showed that the surgery-related death was 17%; they suggested that prosthetic hip surgery was associated with high mortality and morbidity in patients receiving renal replacement therapy. However, the case numbers of these studies [19, 20] and our study are limited. Therefore, further large scale study should be warranted to investigate the association between ESRD and poor outcome in this clinical entity. In contrast, some typical predictors of excess mortality among hip fracture was not found to be associated with age, gender, underlying illness or disease, site of fracture, or type of surgery in this study. This may be explained by the small case number.

In this study, the mortality of patients undergoing early surgery (within 24 hours) was lower than that of patients undergoing delayed surgery (more than 24 hours) (8.6% (7/81) vs 15.0% (3/20), *P* = .410). It is consistent with previous study showed that early surgery improved mortality and morbidity [10]. Moreover, a recent meta-analysis [21] based on 35 independent studies of a total of 191873 patients indicated that delay of surgery would significantly increase in the risk of death. Therefore, early surgery should be encouraged unless further investigation shows negative consequences for early surgery in these clinical conditions.

This study had several significant limitations. The favorable surgical outcome in the present work could be partly due to selection bias. Nonagenarians are supposedly at risk for needing surgery, and only less the one-half of the patients are suitable for the surgery. Thus, a significant portion of patients may be excluded from this study because they did not receive surgery. It indicates that the study subjects in the present work were highly selected. In addition, the data about one year survival or mortality was only available for 75 patients. Because many of the patients who lost of follow-up maybe die, the analysis of one-year mortality may be underestimated in this investigation. Finally, we did not employ a proper outcome measurement, such as questionnaire in this retrospective study. Therefore, we can only analysis the short-term outcome using 30 day- or one year-mortality.

In conclusion, the short-term results for Asian nonagenarians with hip fractures showed that surgical management is a safe intervention in selected patients.
